# Half-Yearly Medical Report of the Hospital for Casual Small-Pox and Vaccination; June 20, 1823

**Published:** 1823-08

**Authors:** George Gregory

**Affiliations:** 8, Upper John-street, Golden-square, London.


Art. II.—*Half-yearly Medical Report of the Hospital for casual Small-Pox and Vaccination; June* 20, 1823.

The proceedings of this establishment during the last six months have not been marked by any circumstances of peculiar interest; they have, nevertheless, been silently working a great deal of public benefit; and, while it must be satisfactory to the Governors to know that their charity has been effectively applied, it may not be unprofitable to the public in general to have their attention re-awakened to the only form of human suffering which mankind universally anticipates.

Eighty-four patients have been admitted into the hospital, between the 19th December, 1822, and the present date. Forty-nine had the disease in a severe and dangerous degree; and of them twenty-three have died. Nine remain under treatment.

Among the thirty-five persons who passed through the complaint in a mild form, twenty-four had been previously vaccinated; and the influence of this process, in stripping the small-pox of its more formidable features, was never more strikingly displayed than in the period now under review. None of them died. While the natural small-pox, indeed, was raging with a degree of violence which hardly admitted of controul by medical aid, those who had been vaccinated remained in comparative security, experiencing no other inconveniences than what result from the visitation of casual disease.

Strongly impressed, by the result of another half-year's experience, with a sense of the benefits of vaccination, your physician is gratified by having to report that, in the same period, 1,605 persons have been vaccinated; and that, with the full knowledge of the occasional failure of this protection, the public voice is still decisively raised in its favour. Your physician cannot, in this place, avoid an allusion to the conduct of some persons, heretofore strenuous advocates of vaccination, who have recently shown a disposition to desert it, because it does not fulfil *all* the expectations which a benevolent, but too sanguine, feeling had once indulged. It behoves such persons, before they recur to inoculation, which experience has proved to be so pregnant with danger to the public welfare, seriously to consider whether the failures of vaccination may not, in some degree, be attributed to causes *under our control*; and whether the alternative proposed may not, even as far as the individual himself is concerned, be as dangerous as the evil to be avoided. Your physician, judging from the experience acquired in this establishment, is disposed to reply to both questions in a manner favourable to the cause of vaccination. He is convinced that a stricter attention than what once was bestowed on the process of vaccination, will assist materially in reducing the number of failures; and he is further of opinion, that the inoculated small-pox is equal in point of severity to the same disease as it occurs casually subsequent to vaccination.

Your physician therefore, in conclusion, again presses on the attention of the Governors the importance of affording every encouragement to vaccination.

GEORGE GREGORY, M.D.

8, *Upper John-street, Golden-square, London.*

---

